# Mechanisms of Network Changes in Cognitive Impairment in Multiple Sclerosis

**DOI:** 10.1212/WNL.0000000000012834

**Published:** 2021-11-09

**Authors:** Danka Jandric, Ilona Lipp, David Paling, David Rog, Gloria Castellazzi, Hamied Haroon, Laura Parkes, Geoff J.M. Parker, Valentina Tomassini, Nils Muhlert

**Affiliations:** From the Division of Neuroscience & Experimental Psychology (D.J., H.H., L.P., G.P., N.M.), School of Biological Sciences, Faculty of Biology, Medicine and Health, University of Manchester, Manchester Academic Health Science Centre, UK; Department of Neurophysics (I.L.), Max Planck Institute for Human Cognitive & Brain Sciences, Leipzig, Germany; Royal Hallamshire Hospital (D.P.), Sheffield Teaching Hospitals, NHS UK; Salford Royal Hospital (D.R.), Salford Royal NHS Foundation Trust, NHS UK; NMR Research Unit (G.C.), Queens Square Multiple Sclerosis Centre, and Centre for Medical Image Computing (G.C., G.P.), Department of Computer Science and Department of Neuroinflammation, Queen Square Institute of Neurology, University College London; Cardiff University Brain Research Imaging Centre (V.T.), Cardiff University, UK; Institute for Advanced Biomedical Technologies (ITAB) (V.T.), Department of Neurosciences, Imaging and Clinical Sciences, University G. d'Annunzio of Chieti-Pescara; and Multiple Sclerosis Centre (V.T.), Department of Neurology, SS Annunziata University Hospital, Chieti, Italy.

## Abstract

**Background and Objectives:**

Cognitive impairment in multiple sclerosis (MS) is associated with functional connectivity abnormalities. While there have been calls to use functional connectivity measures as biomarkers, there remains to be a full understanding of why they are affected in MS. In this cross-sectional study, we tested the hypothesis that functional network regions may be susceptible to disease-related “wear and tear” and that this can be observable on co-occurring abnormalities on other magnetic resonance metrics. We tested whether functional connectivity abnormalities in cognitively impaired patients with MS co-occur with (1) overlapping, (2) local, or (3) distal changes in anatomic connectivity and cerebral blood flow abnormalities.

**Methods:**

Multimodal 3T MRI and assessment with the Brief Repeatable Battery of Neuropsychological tests were performed in 102 patients with relapsing-remitting MS and 27 healthy controls. Patients with MS were classified as cognitively impaired if they scored ≥1.5 SDs below the control mean on ≥2 tests (n = 55) or as cognitively preserved (n = 47). Functional connectivity was assessed with Independent Component Analysis and dual regression of resting-state fMRI images. Cerebral blood flow maps were estimated, and anatomic connectivity was assessed with anatomic connectivity mapping and fractional anisotropy of diffusion-weighted MRI. Changes in cerebral blood flow and anatomic connectivity were assessed within resting-state networks that showed functional connectivity abnormalities in cognitively impaired patients with MS.

**Results:**

Functional connectivity was significantly decreased in the anterior and posterior default mode networks and significantly increased in the right and left frontoparietal networks in cognitively impaired relative to cognitively preserved patients with MS (threshold-free cluster enhancement corrected at *p* ≤ 0.05, 2 sided). Networks showing functional abnormalities showed altered cerebral blood flow and anatomic connectivity locally and distally but not in overlapping locations.

**Discussion:**

We provide the first evidence that functional connectivity abnormalities are accompanied by local cerebral blood flow and structural connectivity abnormalities but also demonstrate that these effects do not occur in exactly the same location. Our findings suggest a possibly shared pathologic mechanism for altered functional connectivity in brain networks in MS.

Cognitive impairment affects about half of people with multiple sclerosis (MS).^1^ Although the disease mechanisms responsible are not fully elucidated, resting-state fMRI (rs-fMRI) studies have shown differences in functional connectivity (FC) between cognitively impaired (CI) and unimpaired patients.^2^ However, a shortcoming of rs-fMRI, which limits the ability to interpret findings, is the lack of information about pathologic mechanisms underlying FC abnormalities.

It has been proposed, in the nodal stress hypothesis, that the high activity of network regions with high connectivity, so-called hubs or nodes, makes them susceptible to pathologic wear and tear, possibly due to high metabolic demands, which could accelerate neurodegeneration, leading to network dysfunction.^3,4^

If wear-and-tear changes are responsible for FC abnormalities, we would expect to see abnormalities also on other magnetic resonance (MR) metrics. Network hubs are heavily interconnected within both functional and structural networks, and activity-related damage can be expected to affect anatomic connectivity. In addition, if nodal damage is caused by unmet metabolic demands, this could affect cerebral blood flow (CBF).^5^ By collecting diffusion MRI and CBF data along with rs-fMRI images, we can establish whether FC abnormalities co-occur with white matter (WM) and perfusion changes, as would be expected under the nodal stress hypothesis. Such co-occurring abnormalities can point to shared underlying mechanisms and thus inform the direction of future research.

In this study, we tested the nodal stress hypothesis in a cohort of patients with relapsing-remitting MS (RRMS) to test whether FC abnormalities in CI patients co-occur with anatomic connectivity and CBF abnormalities in (1) spatially overlapping regions within networks, (2) the same networks, or (3) distal areas from resting-state networks (RSNs).

## Methods

### Participants

One hundred two patients with a diagnosis of RRMS were recruited through the Helen Durham Centre for Neuroinflammation at the University Hospital of Wales, and 27 healthy controls (HC) were recruited from the community. All participants were between 18 and 60 years of age, were right-handed, and had no contraindications for MR scanning. Patients had no comorbid neurologic or psychiatric disease, were relapse-free, and had no change to treatment for 3 months before the MRI scan. All participants underwent MRI scanning and assessment of clinical and cognitive function in 1 study session.

### Standard Protocol Approvals, Registrations, and Patient Consents

The study was approved by the NHS South-West Ethics and the Cardiff and Vale University Health Board R&D committees. All participants provided written informed consent to participate in the study.

### Clinical and Neuropsychological Assessment

Clinical functioning was assessed with the Multiple Sclerosis Functional Composite (MSFC), a standardized measure of upper and lower limb and cognitive function.^6^

All participants underwent neuropsychological assessment with the Brief Repeatable Battery of Neuropsychological Tests, a validated battery with demonstrated sensitivity to cognitive impairment in MS.^7^ Patients' scores on each test were converted to *z* scores with the use of means and SDs from the 27 HC. Patients who scored ≥1.5 SDs below the control mean on ≥2 tests were considered CI, a medium-stringency definition of cognitive impairment.^8^ Remaining patients were considered cognitively preserved (CP). Scores for each of the 4 cognitive domains of verbal memory, visual memory, attention, information processing and executive function, and verbal fluency were calculated by averaging the scores for each test in that domain, as previously described.^8^

### MRI Acquisition

All participants underwent MRI examination on a 3T MR scanner (General Electric HDx MRI System, GE Medical Devices, Milwaukee, WI) with an 8-channel receive-only head radiofrequency coil. A high-resolution 3-dimensional T1-weighted (3DT1) sequence was acquired for identification of T1-hypointense MS lesions, segmentation, registration, and volumetric measurements (resolution 1 × 1 × 1 mm, echo time [TE] 3.0 milliseconds, repetition time [TR] 7.8 milliseconds, matrix 256 × 256 × 172, field of view [FOV] 256 × 256 mm, flip angle 20°). A T2/proton density–weighted sequence (voxel size 0.94 × 0.94 × 4.5 mm, TE 9.0/80.6 milliseconds, TR 3,000 milliseconds, FOV 240 × 240 mm, 36 slices) and a fluid-attenuated inversion recovery sequence (voxel size 0.86 × 0.86 × 4.5 mm, TE 122.3 milliseconds, TR 9,502 milliseconds, FOV 220 × 220 mm, 36 slices) were acquired for identification and segmentation of T2-hyperintense MS lesions. rs-fMRI was acquired with a T2*-weighted gradient-echo echo-planar imaging sequence (voxel resolution 3.4 × 3.4 × 3 mm, TE 35 milliseconds, TR 3,000 milliseconds, FOV 220 × 220 mm, 100 volumes, 46 axial slices, each in an interleaved order), during which all participants were instructed to relax with their eyes closed. Diffusion MRI (dMRI) was acquired with a twice-refocused diffusion-weighted spin-echo echo-planar sequence with 6 volumes with no diffusion weighting and 40 volumes with diffusion gradients applied in uniformly distributed directions (Camino 40) (b = 1,200 s/mm^2^, voxel size 1.8 × 1.8 × 2.4 mm, TE 94.5 milliseconds, TR 16,000 milliseconds, FOV 230 × 230 mm, 57 slices). CBF was quantified with multi-inversion time-pulsed arterial spin labeling (ASL). A PICORE QUIPSS II sequence with a dual-echo gradient-echo readout and spiral k-space acquisition was used (voxel size 3 × 3 × 8 mm, 22 slices).^9^ Sixteen tag-control pairs each for short inversion times (TIs) (400, 500, 600, 700 milliseconds) and 8 tag-control pairs for long TI (1,100, 1,400, 1,700, and 2,000 milliseconds) were acquired with QUIPSS II cutoff at 700 milliseconds. A calibration (M_0_) image was acquired to obtain the equilibrium magnetization of CSF, needed for the quantification of CBF. A minimal contrast image was acquired with TE of 11 milliseconds and TR of 2,000 milliseconds to correct for coil inhomogeneities.

### 3DT1 Image Analysis

Structural 3DT1 images from patients were lesion filled, as described by Lipp et al.^10^, to allow better segmentation of brain tissue, and then segmented into gray matter (GM), WM, and CSF with the FSL Automated Segmentation Tool.^11^ The quality of segmentation was assessed manually. Binary masks of intracranial brain tissue excluding CSF were created from the GM and WM images for use in dMRI analyses. Brain volumes, including whole brain volume, GM volume, and WM volume, were quantified from lesion-filled 3DT1 images with the FSL SIENAX tool.^12^ Lesion volume was calculated from binary lesion masks created as part of lesion filling.

### rs-fMRI Analysis

rs-fMRI blood oxygen level–dependent time series were corrected for physiologic noise in MATLAB^13^ (MathWorks, Natick, MA) with the use of a previously established pipeline.^14^ rs-fMRI images were preprocessed with the FSL MELODIC pipeline,^15^ which included motion correction, spatial smoothing with a 3-mm full width at half-maximum gaussian kernel, high-pass temporal filtering equivalent to 0.01 Hz, nonlinear registration to Montreal Neurological Institute (MNI) standard space, and resampling to a resolution of 4 mm isotropic. Head motion parameter estimates of absolute and relative displacement values did not differ between any groups (HC-RRMS *p* = 0.58 [absolute], *p* = 0.27 [relative]; CP-CI *p* = 0.11 [absolute], *p* = 0.52 [relative]).

Independent component analysis, part of the MELODIC pipeline, decomposed the concatenated dataset into 82 components. Four RSNs that have been found to be important for cognitive function in MS were manually identified and selected for further analyses: the default mode network (DMN),^16,17^ left and right frontoparietal networks (LFPN, RFPN)^16-18^ and the salience network.^17,19^ The anterior and posterior parts of the DMN (DMNa and DMNp, respectively)^20^ were identified in 2 additional components. The primary visual network was used as a noncognitive control network. Dual regression^15^ was used to generate subject-specific versions of the group-average components.

### dMRI Analysis

Preprocessing of dMRI data was carried out in ExploreDTI (version 4.8.3^21^) and included motion correction and corrections for eddy current and echo planar imaging–induced geometric distortions by registering each diffusion image to its respective (skull stripped and downsampled to 1.5 mm) 3DT1 image^22^ with Elastix,^23^ with appropriate reorientation of the diffusion-encoding vectors.^24^ The FSL FDT tool was used to fit diffusion tensors, to generate fractional anisotropy (FA) maps, and to fit the probabilistic diffusion model.^25,26^ Processed diffusion data were quality checked manually. Anatomic connectivity maps (ACMs) were generated with the FSL Probtrackx2 tool^25,26^ by seeding tractography with 50 initiated streamlines per voxel in the binary parenchymal mask. The resulting ACM maps show anatomic connectivity across the whole brain in which the magnitude of the ACM value in each voxel represents the number of probabilistic streamlines passing through that voxel,^27^ thus assessing the degree of anatomic interconnection of every voxel in the brain.^28,29^ Each participant's ACM image was divided by the number of voxels in the brain parenchymal mask to normalize for intracranial volume. To normalize to MNI space, the downsampled 3DT1 image of each participant was nonlinearly registered to MNI space, and the warps were applied to the ACM images.

### ASL Analysis

The 2 sets of ASL tag-control images were motion corrected to the M_0_ image by the FSL McFLIRT tool,^30^ control-tag subtracted, averaged across pairs, and combined into a single multi-TI series that was fed to oxford_asl (BASIL)^31^ for CBF quantification. CBF was estimated with partial volume correction,^32^ coil sensitivity correction (bias field calculated with the SPM12^33^ segmentation on the minimum contrast image), and calibration with the M_0_ signal from participant-specific ventricle masks. CBF maps were then registered to the T1 structural scan following 6 *df* affine registration of the M_0_ scan. T1-weighted images were nonlinearly normalized to the MNI 152 template space with ANTs SyN,^34^ and the obtained warp was applied to the CBF maps. Full CBF maps could not be obtained for all participants due to technical problems with the MR acquisition or due to failed qualitative quality checks of the data. CBF analyses were therefore conducted on data from 49 CI and 43 CP patients. The excluded patients did not differ substantially on demographic and clinical variables from the remaining CI and CP groups.

### Statistical Analyses

Statistical analyses of the demographic, clinical, global MRI, and median ACM, FA, and CBF values were performed in SPSS version 23.0.^35^ The distributions of all variables were checked with Kolmogorov-Smirnov tests and visual inspection of histograms and Q-Q plots. Variables showing a skew were analyzed with nonparametric tests. To test the hypothesis that RSNs that show FC abnormalities also show ACM, FA, and CBF abnormalities, we considered that ACM, FA, and CBF changes could be either in the same voxel clusters that showed FC abnormalities or elsewhere in the affected network. This was tested in analysis steps 1 and 2. In addition, we conducted an exploratory analysis of ACM, FA, and CBF changes throughout the brain to understand how widespread these are in CI compared to CP patients. The data was analyzed as follows.

#### Assessment of Spatially Overlapping Changes

Binary masks of the RSN voxels clusters that showed significant FC differences between the CI and CP groups were created and used to extract local median ACM, FA, and CBF values of these regions, which were then compared between the CI and CP groups.

#### Assessment of Local Changes Within RSNs

Second, we determined whether there were more diffuse changes in anatomic connectivity and CBF throughout each RSN. A binary mask of each RSN was created and, for dMRI analyses, dilated by 1 voxel to include the WM surrounding RSN regions. Voxelwise analyses of ACM, FA, and CBF maps were conducted to look for abnormalities within the RSN regions. For FA, this was done both with skeletonized FA maps in a tract-based spatial statistics (TBSS) analysis^36^ and with nonskeletonized FA maps. TBSS overcomes the difficulties of achieving accurate registration of the WM by projecting all participants’ FA data onto a mean FA tract skeleton before applying voxelwise cross-participant statistics. However, the FA skeleton includes only the center of WM tracts^37^ and may not capture WM local to GM network regions; hence, we conducted both in an exploratory analysis to determine which is most sensitive to FA changes in and around RSNs. Next, we extracted median ACM, FA, and CBF values from the RSNs and compared them between CI and CP patients. The voxelwise analysis approach can show the spatial location of any abnormalities in the metrics studied but requires the abnormalities to be in the same spatial location in most individuals in a group for a group difference to be detected. If this is not the case, a group difference could be missed. Hence, we also extracted median values from our regions of interest in an exploratory analysis. Medians, rather than means, were extracted because ACM, FA, and CBF values were not normally distributed in RSN regions.

#### Diffuse Changes in Anatomic Connectivity and CBF Throughout the Brain

Last, we checked whether the CI and CP groups showed differences in ACM, FA, and CBF throughout the brain by running voxelwise analysis on the ACM, FA, and CBF maps of the whole brain. This was an exploratory analysis to understand the spatial extent of ACM, FA, and CBF abnormalities.

#### Comparisons, Thresholding, and Multiple-Comparison Correction

Comparisons of FC were conducted for both the whole RRMS group with the HC group and the CI and CP patient groups to each other to determine whether FC abnormalities are present in our RRMS cohort and to assess how they differ between the 2 patient subgroups. Subsequent analyses of anatomic connectivity and CBF were conducted only for the 2 patient groups to limit the number of statistical comparisons and in line with our hypotheses.

Comparisons of median ACM, FA values, and CBF values were performed with a 2-sample *t*-test or Mann-Whitney *U* test as appropriate. A Bonferroni correction for multiple comparisons, of a factor of 4 for the 4 RSNs of interest, was applied to the results. The corrected threshold was *p* ≤ 0.0125.

For all voxelwise analyses, age, sex, and education level were included in general linear models as covariates, and all results were threshold-free cluster enhancement corrected at *p* ≤ 0.05, 2 sided. For rs-fMRI analyses, we calculated the percentage of network voxels showing abnormal FC between groups and retained only those RSNs showing the largest proportion of abnormal network voxels for further analyses to reduce the influence of noise. The Harvard-Oxford cortical structural, Harvard-Oxford subcortical structural, and Johns Hopkins University white-matter tractography atlases in FSL were used to report anatomic locations.

### Data Availability

Anonymized data will be shared at the request of other investigators for purposes of replicating procedures and results.

## Results

### Demographic, Clinical, and Neuropsychological Characteristics and Conventional MRI Data

Demographic and clinical characteristics of HC, patients with RRMS, and the CI and CP subgroups are presented in Table 1. Patients with RRMS and controls showed no significant differences in sex, but the RRMS group was significantly older and less educated than the controls and performed worse on all MSFC tests. Fifty-five patients met the definition for CI, and 47 were considered CP. Compared to CP patients, CI patients did not differ significantly in age, sex, education, disease duration, or lower limb function, as measured by the 25-Foot Walk Test of the MSFC. However, their performance on the 9-Hole Peg Test demonstrated worse upper limb function. CI patients showed impaired cognitive function compared to CP patients and HC on all 4 cognitive domains assessed by the Brief Repeatable Battery of Neuropsychological Tests (Table 2). The greatest impairments were observed on the information processing, attention and executive function, and verbal memory domains. CP patients did not perform significantly worse than controls on any domain. Patients with RRMS had significantly lower normalized brain volume and normalized GM volume than HC, but showed no significant difference in normalized WM volume. The CI and CP groups showed no significant differences in any volumetric brain measures (Table 2).

**Table 1:**
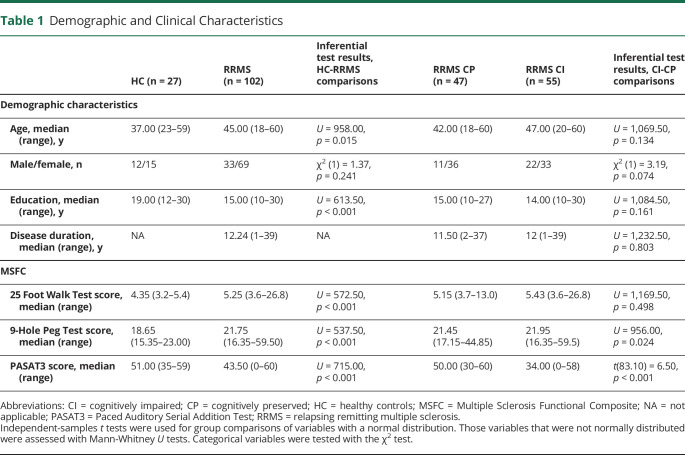
Demographic and Clinical Characteristics

**Table 2:**
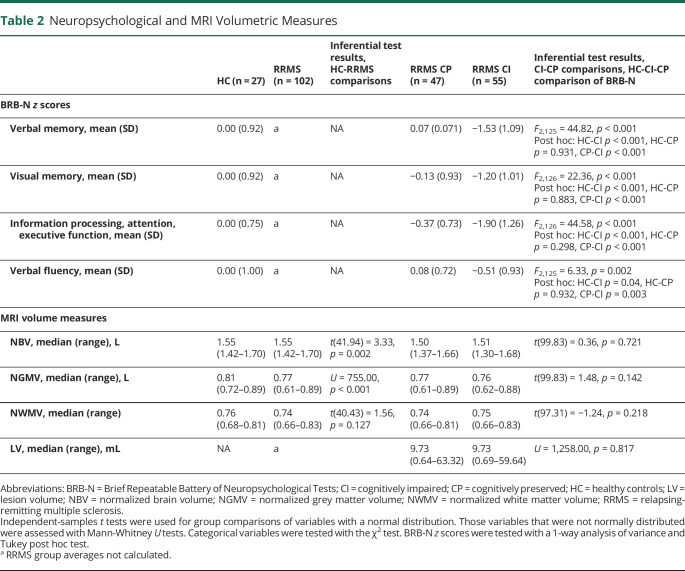
Neuropsychological and MRI Volumetric Measures

### Functional Connectivity

Patients with RRMS showed FC abnormalities in all RSNs investigated compared to HC.

**Figure 1:**
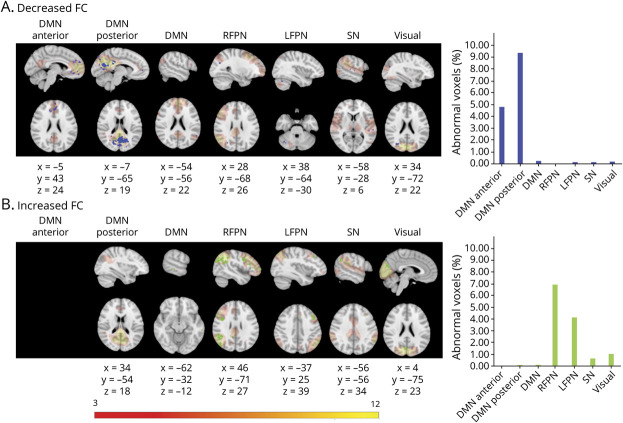
FC Abnormalities in CI Compared to CP Patients Figure shows voxels showing functional connectivity (FC) abnormalities in cognitively impaired (CI) compared to cognitively preserved (CP) patients overlaid onto the group average spatial map of each resting-state network (RSN) analyzed in red-yellow. First 7 columns in each panel show each of the RSNs investigated: default mode network (DMN) anterior, DMN posterior, DMN, right frontoparietal network (RFPN), left frontoparietal network (LFPN), salience network (SN), and primary visual network. For networks not displayed, no significant group differences were found. The 8 columns show graphs indicating the percentage of voxels showing abnormalities of the total number of voxels in the network. Rows show areas of (A) decreased FC in the CI group vs CP (in blue) and (B) increased FC in CI group (in green). Results were threshold-free cluster enhancement corrected at *p* ≤ 0.05, 2 sided. Montreal Neurological Institute coordinates are given for results displayed. Color bar shows signal intensity of RSNs.

CI patients had areas of decreased FC in the DMNa, DMN, DMNp, LFPN, and primary visual network and increased FC in areas of the DMN, salience network, RFPN, LFPN, and primary visual network relative to CP patients. The DMNa, DMNp, LFPN, and RFPN showed the largest proportion of abnormal voxels between groups and were therefore retained for subsequent analyses (Figure 1).

### Anatomic Connectivity and CBF

#### Local Changes in ACM, FA, and CBF in Regions Showing FC Changes

In RSN regions that showed FC changes in CI patients compared to CP patients, there were no significant differences in median ACM, FA, and CBF values between the CI and CP groups after application of a Bonferroni correction for multiple comparisons (corrected *p* threshold = 0.0125).

#### Diffuse Changes in Connectivity Within RSNs

**Figure 2:**
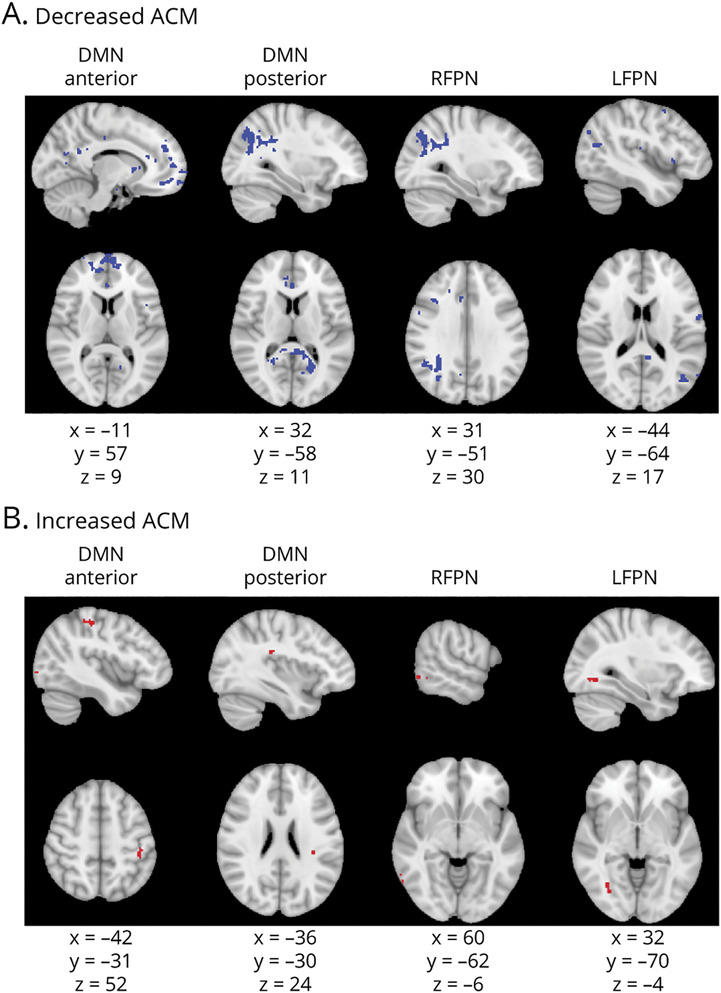
Anatomic Connectivity Changes in Cognitively Impaired Compared to Cognitively Preserved Patients Based on a Voxelwise Analysis of ACMs Figure shows voxels showing anatomic connectivity map (ACM) value abnormalities. Columns show each of the resting-state networks compared. First row (A) shows areas of decreased ACM values (in blue); second row (B) shows areas of increased ACM values (in red). Montreal Neurological Institute coordinates are given for the biggest voxel clusters displayed. Results were threshold-free cluster enhancement corrected at *p* ≤ 0.05, 2-sided. DMN = default mode network; LFPN = left frontoparietal network; RFPN = right frontoparietal network.

Voxelwise analyses of ACM, FA and CBF demonstrated abnormalities in all 4 RSNs in CI compared to CP patients. ACM was reduced in DMNa regions that correspond to the forceps minor, left cingulum, left anterior thalamic radiation, and right anterior corona radiata; DMNp regions, including parts of the splenium of the corpus callosum, left and right cingulum, forceps major, and forceps minor; RFPN WM corresponding to parts of the right inferior longitudinal fasciculus (ILF) and the right inferior fronto-occipital fasciculus; and LFPN regions corresponding to parts of the left superior longitudinal fasciculus, left ILF, and left side of forceps major. There were also areas of increased ACM values, including some voxels in the left superior parietal lobe and left occipital lobe in the DMNa, in a part of the left superior longitudinal fasciculus in the DMNp, the right posterior temporal lobe in the RFPN, and regions of the occipital lobe that could be in either the right ILF or right inferior fronto-occipital fasciculus in the LFPN (Figure 2).

**Figure 3:**
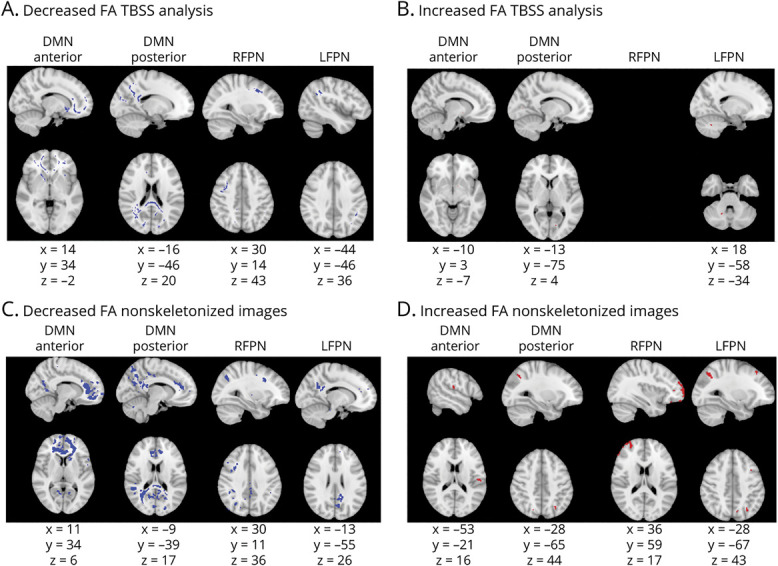
FA Changes in Cognitively Impaired Compared to Cognitively Preserved Patients Figure shows voxels showing fractional anisotropy (FA) abnormalities. (A and B) Results from the tract-based spatial statistics (TBSS) analysis. (C and D) Results from the voxelwise analysis of nonskeletonized FA maps. Columns show each of the resting-state networks compared. First row shows areas of decreased FA (in blue); second row shows areas of increased FA (in red). Montreal Neurological Institute coordinates are given for the biggest voxel clusters displayed. For networks not displayed, no significant results were found. Results were threshold-free cluster enhancement corrected at *p* ≤ 0.05, 2 sided. DMN = default mode network; LFPN = left frontoparietal network; RFPN = right frontoparietal network.

The TBSS analysis showed FA reductions in the genu of the corpus callosum, forceps minor, and cingulum bilaterally in the DMNa; in the splenium of the corpus callosum, posterior parts of the cingulum bilaterally, and posterior corona radiata bilaterally in the DMNp; in parts of the right frontal lobe and right parietal lobe in the RFPN; and in the left side of the splenium of the corpus callosum, left side of forceps major, and left cingulum in the LFPN (Figure 3A). There were also small areas of FA increases across the frontal and parietal lobes (Figure 3B). The voxelwise analysis of nonskeletonized FA maps found FA changes in largely the same regions as the TBSS analysis (Figure 3, C and D).

**Figure 4:**
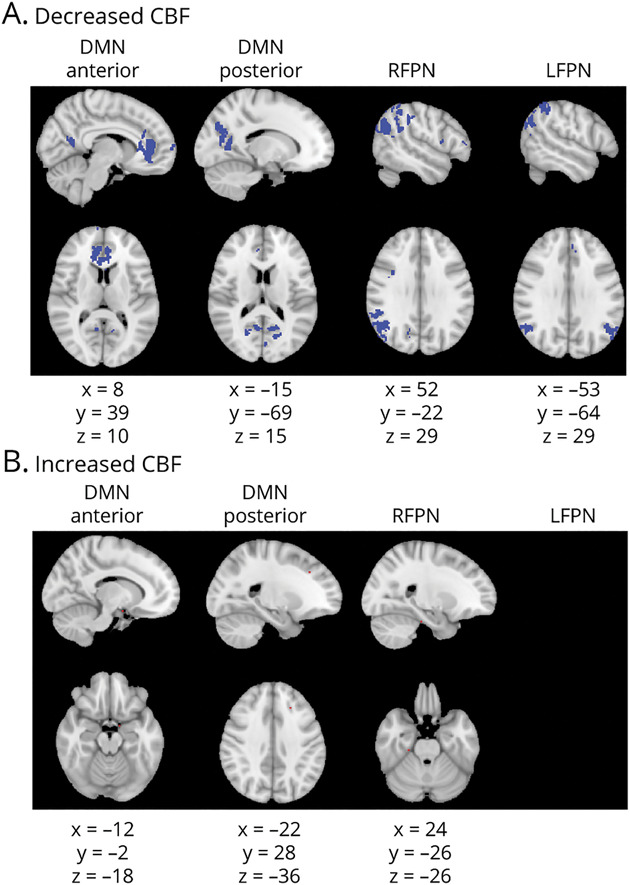
CBF Changes in Cognitively Impaired Compared to Cognitively Preserved Patients Based on a Voxelwise Analysis of CBF Maps Figure shows voxels showing cerebral blood flow (CBF) abnormalities in red. Columns show each of the resting-state networks compared. First row (A) shows areas of decreased CBF (in blue). Second row (B) shows areas of increased CBF (in red). Montreal Neurological Institute coordinates are given for the biggest voxel clusters displayed. Results were threshold-free cluster enhancement corrected at *p* ≤ 0.05, 2 sided. DMN = default mode network; LFPN = left frontoparietal network; RFPN = right frontoparietal network.

There were regions of reduced CBF in all 4 networks in CI compared to CP patients (Figure 4). Reductions were seen in the bilateral cingulate gyrus and precuneus in the DMNa; bilateral precuneus, left cluneal cortex, right lateral occipital cortex, left lingual gyrus, and left posterior cingulate gyrus in the DMNp; and the right occipital cortex, right angular gyrus, right superior supramarginal gyrus, and right cingulate gyrus in the RFPN. The same regions but in the left hemisphere showed CBF reductions in the LFPN. We found some individual voxels, likely artifacts, showing increased CBF in CI patients in the DMNa, DMNp, and RFPN (Figure 4).

Comparisons of extracted median values only found reduced ACM in CI patients (median 0.0039) compared to CP patients (median 0.0043) in the anterior DMN (*U* = 897.00, *p* = 0.008) but no other RSNs. There were no differences in median FA or CBF values in RSN regions.

#### Diffuse Changes in Connectivity and CBF Throughout the Brain: Rationale and Results

**Figure 5:**
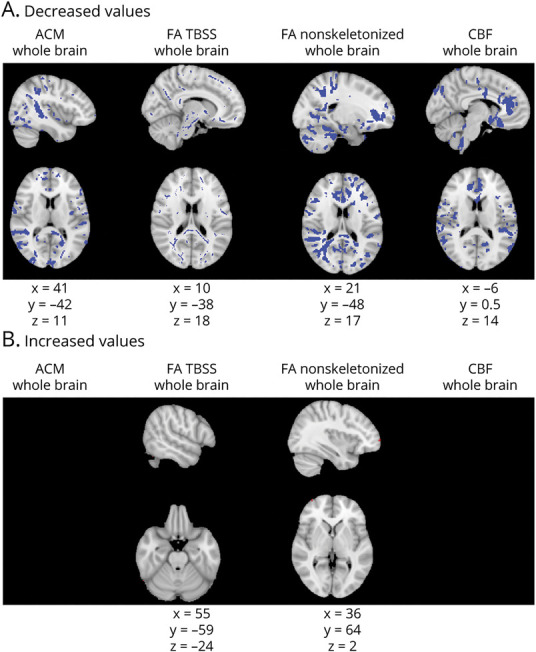
Diffuse ACM, FA, and CBF Changes Across the Whole Brain in CI Compared to CP Patients Figure shows anatomic connectivity map (ACM), fractional anisotropy (FA), and cerebral blood flow (CBF) abnormalities throughout the brain. Columns show each of the metrics assessed: ACM, FA from tract-based spatial statistics (TBSS), FA from analysis of nonskeletonized FA maps, and CBF, in that order. First row (A) shows areas of decreased values (in blue); second row (B) shows areas of increased values (in red). Montreal Neurological Institute coordinates are given for the biggest voxel clusters displayed. Results were threshold-free cluster enhancement corrected at *p* ≤ 0.05, 2 sided.

CI patients compared to CP patients had widespread ACM and FC reductions throughout the brain and some small areas of increased ACM and FC at the edges of the brain. CBF was decreased throughout the brain (Figure 5).

## Discussion

In this study, we provide the first evidence that abnormal FC co-occurs with altered structural connectivity and CBF in CI patients with MS in RSN regions. At the same time, our findings reveal that the exact location of abnormalities differed between metrics. Overall, this indicates that RSNs may be vulnerable to clinically relevant MS pathology, offering partial support for activity-related wear-and-tear damage of network hubs predicted by the nodal stress hypothesis.^3,4^

We found FC abnormalities in our RRMS cohort relative to HC in all RSNs investigated, confirming FC changes as a widespread pathologic feature in MS, as per previous studies.^38^ In CI compared to CP patients, we found FC abnormalities in all networks investigated, with FC decreases in the DMNa and DMNp and increases in the RFPN and LFPN making up the highest proportion of affected network voxels. Increased FC could reflect compensatory mechanisms after structural damage, and decreased FC could be evidence of network breakdown.^39,40^ However, we did not assess the extent of structural damage and can therefore only speculate about the mechanisms of directional FC change, which is an urgent research priority in this field. Nevertheless, our results are consistent with numerous previous reports of abnormal FC in these networks in patients with cognitive symptoms.^16-18^ It is important to note that the FC measure distinguished the 2 patient groups in the absence of significant differences in conventional MR metrics, demonstrating its potential heightened sensitivity to clinically relevant pathology in MS and highlighting the importance of understanding the mechanisms of FC changes.

As predicted, we found reduced anatomic connectivity of networks showing FC abnormalities in CI patients with both the ACM and FA metrics. ACM is an anatomic network measure that shows whether the structural connectivity of a region is affected as a result of WM damage, regardless of where in the brain the WM damage is. It is informative of the degree of connectivity of our regions of interests but not about the WM in and around RSN regions. To understand local tissue characteristics of RSN regions, we also tested the FA metric, a measure of the directionality of diffusion within tissue, which is assumed to be determined by the presence of aligned axons in WM bundles^41^ and can give information about local microstructural integrity in a WM tract. The specific voxels showing FC abnormalities were not those that showed structural changes in CI patients. Instead, other parts of the RSNs were affected. This, combined with widespread ACM and FA changes, suggests that more diffuse, as opposed to focal, anatomic changes within RSNs are associated with cognitive impairment and is in line with previous evidence showing that FC changes are preceded by a high degree of structural damage.^39,40^

In addition to reductions, we found small regions of increased ACM and FA in all 4 RSNs. One possibility is that these are statistical artifacts. ACM increases could reflect an unmasking effect whereby tracking becomes easier in regions where fibers are lost. However, Bozzali et al.^27^ found ACM increases in patients with Alzheimer disease and considered that they may be due to plasticity driven by medication. The mechanism of FA increases is not well understood, but it has been suggested that increased FA reflects changes in axonal structures such as reduced branching, decreased axon diameter, reduced packing density, or increases in myelination.^41,42^ In MS, FA increases may be related to inflammatory processes.^43^ We cannot conclude which mechanisms are responsible for the ACM and FA increases in our CI group but acknowledge the findings as important areas for future research.

Finally, we investigated CBF, which may be a response to decreased energy demand in MS.^5,44^ As with ACM and FA, CBF was reduced in and around RSN regions in CI relative to CP patients but not within the specific voxel clusters showing FC abnormalities, again pointing to diffuse rather than focal tissue abnormalities in RSNs. CBF reductions may reflect a response to decreased energy demand in the RSNs investigated, demonstrating altered metabolic function of RSN regions. However, there are suggestions that CBF changes could be due to a primary vascular insult,^5^ and future studies with more direct measures of metabolism, such as fluorodeoxyglucose PET could help elucidate the metabolic status of functional networks.

Overall, our findings show that diffuse ACM, FA, and CBF abnormalities co-occur with RSN FC changes in CI patients with MS, consistent with the nodal stress hypothesis. The mechanism of nodal wear and tear remains to be elucidated and may relate to unmet metabolic demands.^3,4^ There is preliminary evidence that functional networks are susceptible to metabolic changes, recently from a drosophila model in which network changes are coupled to neuronal metabolism.^45^ Similarly, metabolic changes have been reported in demyelinated axons^46,47^ and if this results in axonal damage or dysfunction that could be reflected in WM metrics in and around affected RSN regions. Thus, our results are not inconsistent with a role of metabolic changes in RSN regions. However, our methods are indirect measures of metabolic function. Other MR modalities such as fluorodeoxyglucose PET support the role of shared metabolic patterns between regions on RSNs,^48^ and 23 Na MRI can show changes in sodium concentration in tissue, which is a measure of the energy state of axons.^44^ If combined with rs-fMRI, these methods may be informative about the metabolic basis of FC changes.

There are limitations to consider when interpreting these results. First, our control group was younger and more educated than our patient cohort. We controlled for this by including age, sex, and education as covariates in our analyses. It is important to note that the CI and CP groups did not differ significantly on these demographic variables. We also did not investigate separate cognitive domains but looked at overall cognition. There have been suggestions that domains may be affected differently by pathology,^49^ and this is an important avenue for future work. Furthermore, we conducted several exploratory analyses to understand how best to explore changes in WM metrics and CBF in and around functional network regions. Comparisons of extracted median ACM, FA, and CBF values from RSN regions showed no group differences between CI and CP patients, pointing to heterogeneity in the metrics across the regions. We conclude that the voxelwise analysis is more sensitive to group differences. The TBSS analysis and the voxelwise analysis of nonskeletonized FA maps showed FA reductions in largely the same regions. The latter additionally showed FA changes at the WM-GM boundaries, which could reflect FA abnormalities in the GM, as has been reported in MS in several studies (reviewed by Inglese and Bester^50^). However, findings of group differences at the edge of the brain and at the midline point to partial volume effects due to registration problems with nonskeletonized FA images and suggest that results need to be interpreted with caution. Related to this, we assessed metrics that are susceptible to partial volume effects. However, the same MR sequences were used for all participants, so any tissue contamination is unlikely to introduce bias in our group comparisons. Finally, we assessed whether FC, ACM, FA, and CBF changes co-occur, but we did not test whether these changes are correlated, which should be investigated in future studies with larger samples.

Our study provides evidence that FC changes in CI patients with RRMS co-occur with abnormal blood flow and anatomic connectivity. This highlights the possibility of a common underlying pathologic change in RSNs such as altered metabolic state in CI patients. The metabolic state of functional networks affected by MS should be further investigated with more direct methods of metabolic brain function to determine the pathologic basis of FC abnormalities and potentially lead to their use as effective biomarkers of disease.

## Publication History

This manuscript was pre-published in medRxiv, doi: https://doi.org/10.1101/2020.11.20.20235309. Received by *Neurology* February 5, 2021. Accepted in final form September 13, 2021.

## Glossary

ACM: anatomic connectivity map
ASL: arterial spin labeling
CBF: cerebral blood flow
CI: cognitively impaired
CP: cognitively preserved
DMN: default mode network
DMNa: anterior part of DMN
DMNp: posterior part of DMN
dMRI: diffusion MRI
FA: fractional anisotropy
FC: functional connectivity
FOV: field of view
GM: gray matter
HC: healthy control
ILF: inferior longitudinal fasciculus
LFPN: left frontoparietal network
MNI: Montreal Neurological Institute
MR: magnetic resonance
MS: multiple sclerosis
MSFC: Multiple Sclerosis Functional Composite
RFPN: right frontoparietal network
RRMS: relapsing-remitting MS
rs-fMRI: resting-state fMRI
RSN: resting-state network
TBSS: tract-based spatial statistics
TE: echo time
TI: inversion time
TR: repetition time
3DTI: 3-dimensional T1-weighted
WM: white matter

## References

[1] Sumowski JF, Benedict R, Enzinger C, et al. Cognition in multiple sclerosis: state of the field and priorities for the future. Neurology. 2018;90(6):278-288. 29343470 10.1212/WNL.0000000000004977 PMC5818015

[2] Benedict RHB. Cognition in multiple sclerosis: Charcot was right. Lancet Neurol. 2020;19(10):810. 10.1016/S1474-4422(20)30306-9 32949540

[3] Buckner RL, Sepulcre J, Talukdar T, et al. Cortical hubs revealed by intrinsic functional connectivity: mapping, assessment of stability, and relation to Alzheimer's disease. J Neurosci. 2009;29(6):1860-1873. 19211893 10.1523/JNEUROSCI.5062-08.2009 PMC2750039

[4] Zhou J, Gennatas ED, Kramer JH, Miller BL, Seeley WW. Predicting regional neurodegeneration from the healthy brain functional connectome. Neuron. 2012;73(6):1216-1227. 22445348 10.1016/j.neuron.2012.03.004 PMC3361461

[5] Lapointe E, Li DKB, Traboulsee AL, Rauscher A. What have we learned from perfusion MRI in multiple sclerosis? AJNR Am J Neuroradiol. 2018;39(6):994-1000. 29301779 10.3174/ajnr.A5504 PMC7410640

[6] Cutter GR, Baier ML, Rudick RA, et al. Development of a multiple sclerosis functional composite as a clinical trial outcome measure. Brain. 1999;122(pt 5):871-882. 10355672 10.1093/brain/122.5.871

[7] Amato M, Portaccio E, Goretti B, et al. The Rao's Brief Repeatable Battery version B: normative values with age, education and gender corrections in an Italian population. Mult Scler. 2006;12(6):787-793. 17263008 10.1177/1352458506070933

[8] Sepulcre J, Vanotti S, Hernández R, et al. Cognitive impairment in patients with multiple sclerosis using the Brief Repeatable Battery-Neuropsychology test. Mult Scler. 2006;12(2):187-195. 16629422 10.1191/1352458506ms1258oa

[9] Warnert EAH, Murphy K, Hall JE, Wise RG. Noninvasive assessment of arterial compliance of human cerebral arteries with short inversion time arterial spin labeling. J Cereb Blood Flow Metab. 2015;35(3):461-468. 25515216 10.1038/jcbfm.2014.219 PMC4348387

[10] Lipp I, Jones DK, Bells S, et al. Comparing MRI metrics to quantify white matter microstructural damage in multiple sclerosis. Hum Brain Mapp. 2019;40(10):2917-2932. 30891838 10.1002/hbm.24568 PMC6563497

[11] Zhang Y, Brady M, Smith S. Segmentation of brain MR images through a hidden Markov random field model and the expectation-maximization algorithm. IEEE Trans Med Imaging. 2001;20(1):45-57. 11293691 10.1109/42.906424

[12] Smith SM, Zhang Y, Jenkinson M, et al. Accurate, robust, and automated longitudinal and cross-sectional brain change analysis. Neuroimage. 2002;17(1):479-489. 12482100 10.1006/nimg.2002.1040

[13] Matlab and Statistics Toolbox Release R2011a. MathWorks Inc: Natick, MA; 2011.

[14] Lipp I, Murphy K, Wise RG, Caseras X. Understanding the contribution of neural and physiological signal variation to the low repeatability of emotion-induced BOLD responses. Neuroimage. 2014;86:335-342. 24128735 10.1016/j.neuroimage.2013.10.015 PMC3898985

[15] Beckmann CF, Mackay CE, Filippini N, Smith SM. Group comparison of resting-state FMRI data using multi-subject ICA and dual regression. Neuroimage. 2009;47(s1):s148.

[16] Meijer KA, Eijlers AJCC, Douw L, et al. Increased connectivity of hub networks and cognitive impairment in multiple sclerosis. Neurology. 2017;88(22):2107-2114. 28468841 10.1212/WNL.0000000000003982

[17] Cruz-Gómez ÁJ, Ventura-Campos N, Belenguer A, Ávila C, Forn C. The link between resting-state functional connectivity and cognition in MS patients. Mult Scler J. 2014;20(3):338-348. 10.1177/1352458513495584 23828871

[18] Louapre CC, Perlbarg V, Garcia-Lorenzo D, et al. Brain networks disconnection in early multiple sclerosis cognitive deficits: an anatomofunctional study. Hum Brain Mapp. 2014;35(9):4706-4717. 24687771 10.1002/hbm.22505 PMC6869493

[19] Rocca MA, Valsasina P, Martinelli V, et al. Large-scale neuronal network dysfunction in relapsing-remitting multiple sclerosis. Neurology. 2012;79(14):1449-1457. 22955126 10.1212/WNL.0b013e31826d5f10

[20] Xu X, Yuan H, Lei X. Activation and connectivity within the default mode network contribute independently to future-oriented thought. Sci Rep. 2016;6:21001. 26867499 10.1038/srep21001 PMC4751480

[21] Leemans A, Jeurissen B, Sijbers J, Jones DK. ExploreDTI: a graphical toolbox for processing, analyzing, and visualizing diffusion MR data. Proc Intl Soc Mag Reson. 2009;17:3537.

[22] Irfanoglu MO, Walker L, Sarlls J, Marenco S, Pierpaoli C. Effects of image distortions originating from susceptibility variations and concomitant fields on diffusion MRI tractography results. Neuroimage. 2012;61(1):275-288. 22401760 10.1016/j.neuroimage.2012.02.054 PMC3653420

[23] Klein S, Staring M, Murphy K, Viergever MA, Pluim JPW. Elastix: a toolbox for intensity-based medical image registration. IEEE Trans Med Imaging. 2010;29(1):196-205. 19923044 10.1109/TMI.2009.2035616

[24] Leemans A, Jones DK. The B-matrix must be rotated when correcting for subject motion in DTI data. Magn Reson Med. 2009;61(6):1336-1349. 19319973 10.1002/mrm.21890

[25] Behrens TEJ, Woolrich MW, Jenkinson M, et al. Characterization and propagation of uncertainty in diffusion-weighted MR imaging. Magn Reson Med. 2003;50(5):1077-1088. 14587019 10.1002/mrm.10609

[26] Behrens TEJ, Berg HJ, Jbabdi S, Rushworth MFS, Woolrich MW. Probabilistic diffusion tractography with multiple fibre orientations: what can we gain? Neuroimage. 2007;34(1):144-155. 17070705 10.1016/j.neuroimage.2006.09.018 PMC7116582

[27] Bozzali M, Parker GJM, Serra L, et al. Anatomical connectivity mapping: a new tool to assess brain disconnection in Alzheimer's disease. Neuroimage. 2011;54(3):2045-2051. 20828625 10.1016/j.neuroimage.2010.08.069

[28] Embleton K, Morris DM, Haroon HA, Lambon Ralph MA, Parker GJ. Anatomical connectivity mapping. Proc Int Soc Magn Reson Med. 2007;15:1548.

[29] Cercignani M, Embleton K, Parker GJM, Bozzali M. Group-averaged anatomical connectivity mapping for improved human white matter pathway visualisation. NMR Biomed. 2012;25(11):1224-1233. 22438202 10.1002/nbm.2793

[30] Jenkinson M, Bannister P, Brady M, Smith S. Improved optimization for the robust and accurate linear registration and motion correction of brain images. Neuroimage. 2002;17(2):825-841. 12377157 10.1016/s1053-8119(02)91132-8

[31] Chappell MA, Groves AR, Whitcher B, Woolrich MW. Variational bayesian inference for a nonlinear forward model. IEEE Trans Signal Process. 2009;57:223-236.

[32] Chappell MA, Groves AR, MacIntosh BJ, Donahue MJ, Jezzard P, Woolrich MW. Partial volume correction of multiple inversion time arterial spin labeling MRI data. Magn Reson Med. 2011;65(4):1173-1183. 21337417 10.1002/mrm.22641

[33] Debernard L, Melzer TR, Van Stockum S, et al. Reduced grey matter perfusion without volume loss in early relapsing-remitting multiple sclerosis. J Neurol Neurosurg Psychiatry. 2014;85(5):544-551. 24039024 10.1136/jnnp-2013-305612

[34] Avants BB, Epstein CL, Grossman M, Gee JC. Symmetric diffeomorphic image registration with cross-correlation: evaluating automated labeling of elderly and neurodegenerative brain. Med Image Anal. 2008;12(1):26-41. 17659998 10.1016/j.media.2007.06.004 PMC2276735

[35] IMB SPSS Statistics for Macintosh, Version 23.0: IBM Corp; 2015.

[36] Smith SM, Jenkinson M, Johansen-Berg H, et al. Tract-based spatial statistics: voxelwise analysis of multi-subject diffusion data. Neuroimage. 2006;31(4):1487-1505. 16624579 10.1016/j.neuroimage.2006.02.024

[37] Smith SM, Kindlmann G, Jbabdi S. Tract-based spatial statistics and other approaches for cross-subject comparison of local diffusion MRI parameters. Brain Mapp. 2015;1:437-464.

[38] Filippi M, Agosta F, Spinelli EG, et al. Imaging resting state brain function in multiple sclerosis. J Neurol. 2013;260(7):1709-1713. 23052604 10.1007/s00415-012-6695-z

[39] Schoonheim MM, Meijer KA, Geurts JJGG. Network collapse and cognitive impairment in multiple sclerosis. Front Neurol. 2015;6:82. 25926813 10.3389/fneur.2015.00082 PMC4396388

[40] Tewarie P, Steenwijk MD, Brookes MJ, et al. Explaining the heterogeneity of functional connectivity findings in multiple sclerosis: an empirically informed modeling study. Hum Brain Mapp. 2018;39(6):2541-2548. 29468785 10.1002/hbm.24020 PMC5969233

[41] Beaulieu C. The basis of anisotropic water diffusion in the nervous system - a technical review. NMR Biomed. 2002;15(7-8):435-455. 12489094 10.1002/nbm.782

[42] Hoeft F, Barnea-Goraly N, Haas BW, et al. More is not always better: increased fractional anisotropy of superior longitudinal fasciculus associated with poor visuospatial abilities in Williams syndrome. J Neurosci. 2007;27(44):11960-11965. 17978036 10.1523/JNEUROSCI.3591-07.2007 PMC6673356

[43] Calabrese M, Rinaldi F, Seppi D, et al. Cortical diffusion-tensor imaging abnormalities in multiple sclerosis: a 3-year longitudinal study. Radiology. 2011;261(3):891-898. 22031708 10.1148/radiol.11110195

[44] Paling D, Golay X, Wheeler-Kingshott C, Kapoor R, Miller D. Energy failure in multiple sclerosis and its investigation using MR techniques. J Neurol. 2011;258(12):2113-2127. 21660561 10.1007/s00415-011-6117-7

[45] Mann K, Deny S, Ganguli S, Clandinin T. Causal coupling between neural activity, metabolism, and behavior across the drosophila brain. Nature. 2021;593(7858);244-248. 33911283 10.1038/s41586-021-03497-0 PMC10544789

[46] Craner MJ, Newcombe J, Black JA, Hartle C, Cuzner ML, Waxman SG. Molecular changes in neurons in multiple sclerosis: altered axonal expression of Nav1.2 and Nav1.6 sodium channels and Na+/Ca2+ exchanger. Proc Natl Acad Sci USA. 2004;101(21):8168-8173. 15148385 10.1073/pnas.0402765101 PMC419575

[47] Foster RE, Whalen CC, Waxman SG. Reorganization of the axon membrane in demyelinated peripheral nerve fibers: morphological evidence. Science. 1980;210(4470):661-663. 6159685 10.1126/science.6159685

[48] Savio A, Fünger S, Tahmasian M, et al. Resting-state networks as simultaneously measured with functional MRI and PET. J Nucl Med. 2017;58(8):1314-1317. 28254868 10.2967/jnumed.116.185835 PMC6944183

[49] Migliore S, Ghazaryan A, Simonelli I, et al. Validity of the Minimal Assessment of Cognitive Function in Multiple Sclerosis (MACFIMS) in the Italian population. Neurol Sci. 2016;37(8):1261-1270. 27095052 10.1007/s10072-016-2578-x

[50] Inglese M, Bester M. Diffusion imaging in multiple sclerosis: research and clinical implications. NMR Biomed. 2010;23(7):865-872. 20882528 10.1002/nbm.1515 PMC3071990

